# The Impact of Screen Time on the Health of the Pediatric Population: Short- and Long-Term Consequences for Lifestyle, Ophthalmology, and Mental Health

**DOI:** 10.7759/cureus.96944

**Published:** 2025-11-16

**Authors:** Mateusz Piszka, Eliza Kwapien, Patryk Brasse, Karolina Staszkiewicz, Julia Zerdka, Kacper K Staszkiewicz, Jakub Bartkowski, Filip Czarnecki, Maria Kubicka

**Affiliations:** 1 General Practice, Wojewódzki Szpital Specjalistyczny nr 5 im. św. Barbary w Sosnowcu, Sosnowiec, POL; 2 Orthopedics, SP ZOZ MSWiA w Katowicach im. Sierżanta Grzegorza Załogi, Katowice, POL; 3 General Practice, Miejskie Zakłady Opieki Zdrowotnej w Żorach, Żory, POL; 4 General Practice, Szpital Miejski w Siemianowicach Śląskich, Siemianowice Śląskie, POL; 5 General Practice, Samodzielny Publiczny Szpital Kliniczny im. Andrzeja Mielęckiego w Katowicach, Katowice, POL

**Keywords:** digital eye strain, mental wellbeing, myopia progression, obesity, pediatric, screen exposure time

## Abstract

This review analyzes extensive scientific data linking screen time to a wide range of health problems in the pediatric population and divides them into short-term and long-term health effects. Excessive exposure to screen media negatively affects many aspects of the health of children and adolescents. We can observe negative effects that translate into patients' lifestyles as well as ophthalmological and mental health problems. After analyzing the studies, the most common short-term consequences, such as obesity, sleep disorders, and dry eye syndrome, were identified. Many studies have highlighted the impact of particularly long periods of screen time, more than 3-4 hours. The health consequences that manifest themselves later include hypertension, cardiovascular disease, and myopia. This impact also extends to mental health, where there is a clear correlation between long screen time and a higher incidence of anxiety, depression, and behavioral problems. Evidence indicates an increase in children's screen time since the COVID-19 pandemic. Due to the continuing high level of screen time, associated not only with entertainment but also with the transfer of certain activities to an online format, despite the end of the pandemic, public health interventions and educational strategies are urgently needed to mitigate these negative effects and promote a healthy lifestyle among children, which will translate into the health of the entire population in the future.

## Introduction and background

In recent years, the use of digital devices has become an integral part of childhood, and exposure to screen media is commonplace in the daily lives of children and adolescents [1]. While these technologies provide numerous educational and social opportunities, there is growing evidence linking prolonged screen time to negative health effects in children and adolescents [2]. The rise of digital devices has been linked to a broad spectrum of health challenges, extending beyond traditional physical health metrics to include mental, behavioral, and ophthalmological domains [3-6]. This review synthesizes the current scientific understanding of the relationship between screen time and a child's health. We explore key areas such as the association with lifestyle diseases like obesity and metabolic disorders, as well as the effects on sleep patterns and dietary habits [4,7-13]. Furthermore, we delve into the less-examined but increasingly critical areas of ophthalmological problems, including digital eye strain and myopia, and the profound impact on mental health, such as anxiety, depression, and behavioral issues [14-16]. By examining these multifaceted relationships, this paper aims to provide a comprehensive overview for clinicians, public health professionals, and parents, highlighting the need for targeted interventions and informed guidelines to mitigate the adverse effects of excessive screen time and promote a healthier future for the pediatric population.

## Review

Methodology

We conducted a comprehensive review of peer-reviewed literature published between 2017 and 2025, focusing on studies that investigated the association between screen time and health issues in children and adolescents. The key areas of interest included obesity, sleep disturbances, dietary habits, metabolic disorders, ophthalmological problems, and mental health issues. The text has been edited by an LLM for better comprehension and clarity. The authors maintain full intellectual responsibility for all scientific content and conclusions presented.

Lifestyle-related short-term effects of screen time

Obesity has been recognized as one of the most well-documented consequences of screen media exposure, a relationship that was already emphasized in studies published as early as 2017 [7]. Numerous observational studies have confirmed an association between screen time and an increased risk of overweight, obesity, and non-communicable diseases [3]. An American study conducted in 2017 demonstrated that children spending a significant portion of the day using screen devices exhibited a higher prevalence of overweight, obesity, and other metabolic disorders [7]. Between 2020 and 2021, a marked increase was observed in the number of publications investigating the impact of screen media exposure on children’s health. A noticeable upward trend was observed when comparing research conducted across the same countries and regions before and after the COVID-19 pandemic [11]. What is often perceived as a harmless form of entertainment may, in fact, have a profound influence on children’s development, behavior, and lifestyle. Current evidence suggests that the use of screen media contributes to obesity through several mechanisms, including increased food intake during viewing, exposure to advertisements promoting high-calorie and nutrient-poor foods, reinforcement of unhealthy eating habits, and shortened sleep duration [7].

Sleep Disturbances Associated With Screen Use

A Japanese study conducted in 2019 among children under five years of age found that excessive screen time was associated with shorter sleep duration and poorer sleep quality. Reduced sleep time was, in turn, correlated with longer screen exposure, exacerbating circadian rhythm disturbances [3]. Adequate sleep duration during childhood is essential for cognitive, emotional, and somatic development. Similar findings were reported in a 2018 study involving 177 children aged 8-17 years, where insufficient sleep duration was associated with excessive screen use and an increased risk of overweight [10]. Research conducted during the COVID-19 pandemic (2020-2022) highlighted further increases in screen time, which were associated with intensified sleep disorders, weight gain, hypertension, and the persistence of unhealthy eating patterns [8].

Dietary Habits and Eating Behaviors

The relationship between screen time and children’s dietary habits has been extensively described in the literature. A study conducted in Greece demonstrated that with an increasing number of television viewing hours, the frequency of soft drink and fast-food consumption also increased. A statistically significant association was found between total screen time (television, video games, smartphones) and lower adherence to the Mediterranean diet [13]. Greater exposure to advertisements for energy-dense foods additionally intensified children’s purchase requests and shaped their food preferences and consumption behaviors [7]. In the study by Jones et al., excessive screen time (≥2 hours per day) correlated with overweight and sleep disturbances among children aged 0-18 years [9]. Further analyses indicate that children spending more than 3 hours per day in front of screens have a 1.37-fold higher risk of obesity compared to those using screens for less than 1 hour [12]. Similarly, exposure of ≥4 hours per day was associated with a 1.68-fold higher risk of obesity relative to less than 2 hours. Children who watched television for ≥180 minutes daily were 2.86 times more likely to be obese compared with those who watched for less than 60 minutes [17]. A similar association was found for smartphone use (OR [95% CI] = 2.75 [2.06-3.68]) [18].

Lifestyle-related long-term effects of screen time

Both obesity and excessive screen time are independently and synergistically associated with the development of hypertension in adolescents. In one study, adolescents who were obese and reported long screen time had the highest prevalence of hypertension (52.4%), significantly exceeding rates observed in individuals with only one of these risk factors [4].

Metabolic and Cardiovascular Diseases

In a 24-year prospective cohort study (1994-2019) involving 7,105 adolescents aged 11-18 years, each additional hour of daily screen time was associated with an increase in BMI of 0.06 units and with a higher risk of obesity and diabetes in adulthood. At baseline, the mean BMI of participants was 22.57 kg/m², which increased to 30.27 kg/m² over the observation period. By the end of the follow-up, 43.4% of participants were obese, 8.4% had diabetes, 31.8% had hypertension, and 14.9% presented with hyperlipidemia [19]. Although no direct association between screen time and hypertension was observed, a strong relationship between longer exposure and the development of obesity and metabolic disorders was identified. Other studies have confirmed that prolonged screen time during adolescence and its increase in early adulthood are associated with higher cardiometabolic risk. Individuals who increased their screen time by more than two hours per day exhibited higher BMI values and more adverse metabolic parameters [20].

Ophthalmological short-term effects of screen time

Dry eye syndrome (DES), a prevalent heterogeneous disorder of the ocular surface, markedly diminishes the individual's functional quality of life [21,22]. Prolonged daily use of digital display devices was identified as a significant risk factor for a range of ocular and systemic symptoms. Headaches were significantly associated with more than three hours of daily screen time. Furthermore, over four hours of use was linked to a higher incidence of eye pain, foreign body sensation, and lacrimation. Eye fatigue and redness were observed to be significant risks with over five hours of daily exposure [23]. Limiting digital screen exposure is a crucial strategy for mitigating headache symptoms in the pediatric and adolescent population [24]. The most significant correlation identified between digital screen use and headaches was the duration of exposure, with computer use emerging as the most prevalent device type associated with this symptom [25].

Digital Screen Use as a Risk Factor for Dry Eye in Children

Digital eye strain (DES), also known as computer vision syndrome (CVS), is a widespread problem. It's becoming increasingly common globally because more and more people are spending significant time looking at digital screens. Essentially, it's a condition affecting a huge number of individuals who use computers, phones, and tablets regularly. Increased digital screen time in children has been identified as a significant risk factor for the development of digital eye strain (DES) symptoms. Observational studies have consistently shown that prolonged digital screen exposure is a key risk factor for the development of digital eye strain (DES) in both adult and pediatric populations. Furthermore, an elevated risk was noted in individuals who favored smartphones over other devices, in male patients, and in those over 14 years of age [14]. The findings reported by Shah et al. revealed that the majority of participants (479, mean age was 6.7) experienced common symptoms associated with digital eye strain. Specifically, a high prevalence of self-reported headaches or eye pain (78.3%), unhabitual blinking (73%), blurred vision (74%), and foreign body sensation or itching (64.5%) was noted. The study also highlighted that over half of the subjects (55.3%) utilized dedicated digital devices, a factor that may have contributed to the observed symptom profile [15]. Prolonged exposure to mobile screens, defined as two to three hours or four or more hours daily, was directly correlated with a significantly higher incidence of dry eye disease (DED) compared to shorter exposure durations [16]. DED poses a substantial burden on both affected individuals and public health systems. The anticipated rise in the incidence and burden of DED in pediatric patients is likely linked to the widespread use of electronic screens and lifestyle shifts resulting from the COVID-19 pandemic. These changes may have a negative impact on children's mental health and academic performance [26].

Ophthalmological long-term effects of screen time

A significant association was found between screen time exposure and the development of myopia in children and adolescents. Furthermore, evidence suggests that computer use may exert the most pronounced effect on the condition [5]. The prospective cohort study, "The association between smartphone use and myopia progression in children," included a sample size of 523 children, aged 6 to 14 years. It was reported that, over the 24-month study period, the spherical equivalent refractive error showed significant myopic progression. The mean refractive error increased from -0.93 ± 0.52 diopters at 6 months to -1.32 ± 0.65 diopters at 24 months. Concurrently, the mean axial length of the globe correspondingly increased from 23.48 ± 1.25 mm at baseline to 24.00 ± 1.35 mm by the study's conclusion. The rate of myopic progression was substantially higher in children who spent over four hours a day on smartphones, with a mean annual progression of 0.66 ± 0.27 D. This rate was significantly greater than that observed in children with moderate (2-4 h/day, 0.43 ± 0.20 D/year) or minimal (<2 h/day, 0.32 ± 0.16 D/year) screen time [27]. Findings from the linear dose-response meta-analysis demonstrated that even a modest increase of one hour in daily screen time carries a substantial clinical risk, correlating with a 21% higher probability of myopia onset [28].

Psychiatric short-term and long-term effects of screen time

Total screen time, both on weekdays and weekends, has a moderate association with an increased prevalence of behavioral problems. These issues include attention-deficit/hyperactivity disorder (ADHD), suboptimal academic performance, and poor sleep quantity and quality [29]. Based on the research by Cartanyà-Hueso À et al., Spanish children between the ages of 6 and 14 who engaged in at least 180 minutes of daily leisure screen time were found to have a higher risk of developing emotional and behavioral problems compared to their peers who had up to 59 minutes of daily screen time. Specifically, this increased risk was associated with emotional symptoms, peer relationship issues, conduct problems, and social behavior difficulties [6]. In the paper titled "Associations between screen time and emotional and behavioral problems among children and adolescents in the US," the crude model revealed that in populations exposed to more than two hours of daily screen time, excessive screen use was associated with emotional symptoms, conduct problems, peer relationship problems, and total difficulties. This study indicates a significant correlation between prolonged screen exposure and a range of behavioral and emotional challenges in pediatric and adolescent populations [30].

Anxiety Issues

Active social media engagement has been shown to be a predictor of subsequent anxiety symptoms in adolescents. Additionally, frequent digital screen time (defined as three or more hours daily) is associated with a decrease in well-being, specifically impacting a patient's external and prosocial functioning [31]. Prolonged screen time has been clearly associated with the prevalence of anxiety-related disorders. A study of adolescents in Saudi Arabia demonstrated that those with extended screen time during the COVID-19 pandemic were significantly more likely to experience anxiety and suffer from anxiety-related disorders [32].

Major Depressive Disorder in Childhood

Screen media activity (SMA) has been consistently linked to depressive and anxiety disorders and their symptomatology. Research indicates that increased screen time during childhood is directly correlated with a higher incidence of internalizing symptoms in subsequent years. Furthermore, prolonged screen exposure, specifically, more than one hour daily of electronic gaming or computer use and more than two hours daily of television watching, has been shown to be associated with reduced life satisfaction among adolescents [31]. Escapism and avoidant coping strategies are closely linked to problematic screen use and problematic internet use (PUI). This is supported by the widely accepted notion that digital screens possess addictive properties. Individuals who engage in extensive screen time tend to report a greater susceptibility to addiction, a higher prevalence of internalizing symptoms (such as depression and anxiety), and less effective utilization of coping strategies when faced with life stressors. In the Swedish adolescent population, high levels of leisure screen time may impede problem-focused coping mechanisms, thereby contributing to a gradual increase in depressive symptoms over time [33]. Francisquini et al. found a positive correlation between screen time and the presence of depressive, anxiety, and stress symptoms in adolescents. Their findings also showed that adolescents with more than four hours of daily screen time exhibited a higher incidence of common mental health disorders compared to those with less than two hours [34].

Sleep Problems in the Context of Mental Health

Excessive screen time can negatively affect sleep health through multiple physiological and behavioral mechanisms. First, extended use of screens can displace essential sleep time or sleep-promoting activities, such as physical exercise. Second, the stimulating nature of on-screen content and social engagement, particularly for pediatric populations, can create a state of cognitive arousal that impedes the ability to initiate sleep. Finally, the blue light emitted from screens can disrupt the body's natural circadian rhythm by suppressing melatonin production, a hormone critical for sleep-wake cycle regulation [35]. A study by Yara Alshoaibi et al. found a significant inverse relationship between daily screen time and sleep quality among adolescents. A notable 62.2% of adolescents who used screens for more than 8 hours daily were identified as poor sleepers, compared to 59.3% of those with 6-8 hours of use and just 20% of those with less than 1 hour (P=0.001). Furthermore, the research revealed that using a device for more than 60 minutes while in bed before sleep was strongly associated with poor sleep. Specifically, 63.5% of adolescents who did this were poor sleepers, whereas only 35.6% of those who limited their pre-sleep device use to 15 minutes or less were classified as such (P=0.001) [36]. In the study titled "The Impact of Screen Time on Sleep Patterns in School-Aged Children: A Cross-Sectional Analysis," a significant inverse relationship was observed between screen time and sleep efficiency. Children with low screen time demonstrated a significantly higher sleep efficiency (90%) compared to the high screen time group (75%, p<0.01). Furthermore, this research noted that reduced screen time was correlated with fewer nocturnal awakenings (0.5 vs. 1.5 per week) and a lower prevalence of daytime sleepiness (20% vs. 60%) [37].

Interventions aimed at reducing screen time

In 2022, guidelines addressing the management of obesity in adolescents with mental health disorders recommended limiting screen time as part of therapeutic strategies [8]. However, not all studies confirm the effectiveness of such interventions. A meta-analysis conducted the same year revealed that, although interventions focused on reducing screen time can effectively shorten its duration (MD: -6.90 h/week; 95% CI: [-9.19 to -4.60]; p < 0.001), they do not consistently result in significant reductions in BMI [38]. A prospective study from New Zealand investigated the effects of implementing Family Screen Rules (FSR) in households with children aged two years on obesity-related behaviors at 45 months and obesity at 54 months. The study found that adherence to FSR was not directly associated with lower BMI but had a protective effect on obesity-related behaviors by improving sleep duration and reducing excessive screen time. A significant indirect association through health-related behavioral mediation was observed [39].

Discussion

This review synthesizes compelling evidence published between 2017 and 2025, confirming that prolonged screen time represents a significant, multifaceted public health threat to children and adolescents. The findings demonstrate a clear consistency across multiple health domains: excessive digital device use contributes substantially to metabolic dysfunction, driven by sedentary behavior and poor dietary choices, and accelerates long-term conditions like obesity and hypertension [7,19]. Furthermore, critical developmental areas are severely affected, with screen time disrupting sleep hygiene through both blue light exposure and cognitive stimulation, while significantly increasing the incidence and progression of ophthalmological issues such as digital eye strain and myopia [23,27]. The strong correlation observed between high screen use and an increased prevalence of behavioral problems, anxiety, and depressive symptoms underscores the profound mental health burden [6,34]. Collectively, the data highlight that exceeding the threshold of two to four hours of daily screen time is critically linked to adverse outcomes, positioning screen media exposure as a primary, modifiable risk factor requiring urgent public health intervention. The full scope of this critical dose-response relationship is detailed in Table 1.

**Table 1:** Summary of key findings and risks concerning screen time in the pediatric population

Health outcome category	Identified risk	Critical screen time threshold	References
Obesity and metabolism	Increased risk of overweight, obesity, and metabolic disorders	≥3 hrs/day	[7,12]
	Obesity risk 1.37-fold higher	≥3 hrs/day	[12]
	Each additional daily hour associated with a 0.06 unit increase in BMI in adulthood	1 hour/day	[19]
Sleep disturbances	Shorter sleep duration and poorer sleep quality; disruption of circadian rhythm	≥1 hour in bed before sleep	[3,10,36]
	62.2% of adolescents using screens >8 hrs/day identified as poor sleepers	>8 hrs/day	[36]
Ophthalmological (DES)	Digital Eye Strain (DES), headaches, eye pain, fatigue, lacrimation	>3 hrs/day (headaches), >4 hrs/day (eye pain, lacrimation)	[14,23]
Ophthalmological (myopia)	Significant and accelerated progression of myopia (nearsightedness)	>4 hrs/day (smartphone)	[27]
	21% higher probability of myopia onset	1 hour/day	[28]
Behavioral problems	Increased prevalence of emotional, behavioral, and peer relationship problems	≥3 hrs/day (leisure screen time)	[6]
Anxiety and depression	Higher incidence of anxiety and depressive disorders; reduced well-being	≥3 hrs/day (anxiety); >4 hrs/day (common mental health disorders)	[31,34]

Future recommendations

Based on the compelling evidence, future research must move beyond simply establishing the link between screen time and health. The next critical step is to develop and rigorously test a wide range of interventions aimed at mitigating these negative effects. There is a pressing need for multi-component strategies that address not only screen time reduction but also concurrently promote healthier lifestyles, including increased physical activity and better dietary habits. Future studies should focus on implementing and evaluating long-term, community-based programs that involve parents, educators, and healthcare providers. Additionally, a deeper understanding of the specific content and context of screen use is essential. Instead of a blanket approach, researchers should investigate which types of media are most harmful and how the social environment, such as co-viewing with family, can moderate outcomes. Ultimately, the goal is to develop and implement scalable, evidence-based public health policies that can be widely adopted to ensure that the digital age benefits children's well-being rather than undermining it.

## Conclusions

The body of evidence reviewed in this study demonstrates a compelling link between extensive screen time and a range of adverse health outcomes in the pediatric population. The findings consistently show that prolonged digital device use is not merely a benign form of entertainment but a significant contributor to a wide array of health challenges. These include not only a heightened risk of obesity and related metabolic disturbances but also a substantial negative impact on sleep hygiene, vision, and mental well-being. The data reveal a clear association between increased screen exposure and a greater prevalence of behavioral and emotional difficulties.

The evidence highlights the detrimental effects of excessive screen time on children's health, underscoring its role as a key modifiable risk factor. Public health interventions and educational strategies are urgently needed to mitigate these negative impacts and promote healthier lifestyles in the pediatric population. Moving forward, a collaborative effort is essential, involving healthcare providers, educators, parents, and policymakers, to develop and implement effective guidelines. Future research should focus on optimizing these interventions and exploring long-term outcomes to ensure that the digital age benefits, rather than harms, the next generation.
